# Diversity and practice: local decision-making practices on multi-cultural diets for British Muslim school children (BMSC) and implications for social justice

**DOI:** 10.3389/fpubh.2024.1430852

**Published:** 2024-08-05

**Authors:** Zeibeda Sattar, Susan M. Carr, Lydia Lochhead, Margaret Anne Defeyter

**Affiliations:** Department of Social Work, Education and Community Wellbeing, Faculty of Health and Life Sciences, Northumbria University, Newcastle upon Tyne, United Kingdom

**Keywords:** school meals, halal, food caterers, decision-makers, cultural, religious foods

## Abstract

**Introduction:**

British Muslim School Children (BMSC) are required to follow special halal dietary requirements in accordance with their religion, which is often not accounted for in British schools. This often leaves BMSC limited to a vegetarian diet while at school, despite this not being their chosen diet or preference. This study explores the perceptions of key stakeholders regarding fairness and accessibility of school meals for BMSC, as well as discussing school food provision for those maintaining a religious diet in light of social justice. This is in the context of limited knowledge previously being explored in the North East of England regarding procurement and decision-making at a systems level to cater for BMSC.

**Methods:**

A qualitative research design was conducted. A total of 62 participants (39 BMSC, 15 parents, and 8 school and catering staff) took part in a semi-structured interview or focus group. Participants were recruited from six schools, with these schools selected based on their differing levels of BMSC in attendance. This project took place between March 2022 and October 2023.

**Results/discussion:**

Results suggested that where schools already catered for diverse food requirements, inclusive of BMSC dietary needs, food choices were still limited in the options and amount available. School and catering staff stated that cost implications contributed to their menu development process. Despite this, there was an evident willingness to learn about the cultural food options and how these can be implemented in future school menus. Suggestions discussed included an increase in the use of halal meat in order to provide a more inclusive school food experience for BMSC.

## Introduction

### Multi-cultural diversity and school meal provision

Multi-cultural diversity is a worldwide phenomenon, with 3.4% of people worldwide residing in a country other than that of their birth, including 16.8% of the UK population (1–3). The 2021 Census revealed that after the ethnic group of White, the next most common ethnic group was Asian British or Asian Welsh (9.3%; 5.5 million), an increase in comparison to 2011 figures (up 7.5%; 4.2 million). At a local level, from 2011 to 2021, there was an increase from 13.4 to 17.4% of people born outside of the UK residing in Newcastle upon Tyne, a city in the North East of England, with 9% (26,896) of the population in Newcastle upon Tyne identifying as Muslim (1). This large increase in a diverse population means that there is a need for school food to reflect the cultural and religious requirements of its pupils and the local population. Prior research suggests that the limited options available for halal food at school may result in BMSC often consuming a vegetarian diet while at school, despite this not being their regular or chosen diet (4). while schools have a choice regarding school food menus, there is a paucity of research available for school leaders to make informed choices regarding the inclusion of halal food options (5).

Healthy eating is promoted by the UK government, with schools encouraged to provide pupils with healthy, tasty, and nutritious school meals. Compliance with the School Food Standards is a mandatory requirement for all grant-maintained schools, including academies and free schools (6). The School Food Standards set out by the government are there to ensure that (1) the food provided to pupils is nutritious and of high quality, (2) that good nutritional health for pupils is being promoted, and (3) to protect those who are nutritionally vulnerable. The standards highlight the importance of ensuring that pupils are receiving a healthy and balanced diet, with this defined as one consisting of plenty of fruit and vegetables; unrefined starchy food; meat, fish, eggs, or beans; dairy and non-dairy sources of protein; and with minimum amounts of fat, sugar, and salt (6). It has been argued that school meals have been vital in reducing dietary inequalities for pupils (7, 8), with school meals found to be a healthier option compared to a packed lunch regardless of the pupils’ socio-economic class (9, 10). The School Governing Board is responsible for ensuring that the national School Food Standards (SFS) are met in schools located in England. According to the SFS (6), where food is provided by the local authority or a private caterer, compliance with the standards should be specified within the catering contract, with the caterer required to provide the governing body with evidence of this compliance. The School Governing Board is also strongly encouraged to work with the senior leadership team of the school to develop a school food policy strategy regarding the promotion of eating a school-provided meal (6). The SFS outlines that school principals should ensure that their school meals provide familiar food as well as new food that pupils may not have tried before (6). Local authorities follow national guidance provided by the SFS, with menus and daily selections varying from school to school (11). In light of this, the SFS encourages schools to make reasonable adjustments for pupils who have particular dietary requirements (6).

### Food justice

Food justice is the collective right to a fair food system through developing individual and community empowerment, dignity, and political representation (12). Although religious needs are not outlined in the SFS, fairness and access to opportunities are key to social justice within a society (13), and the omission may lead to exclusionary practice and poor dietary intake.

### Halal school food

Halal food is defined in the Qur’an as the ritual slaughtering of animals adhering to Islamic Law. Meat that is produced and does not follow Islamic Law is defined as haram food, meaning in Arabic that it is forbidden for Muslims to eat (14). Halal and haram are key in the life of a Muslim, with these directing aspects of their lives such as the use of devotional practices, their finances, marital issues, and health (15). Where schools do not offer halal food, it has been argued by Islamic leaders that the refusal to provide halal food is discriminatory (16), with British Muslim School Children (BMSC) unable to practice their religious rights and prioritize taste preference in the school dining hall. There have been issues noted in terms of what alternative food is available to those practicing religions, with some Muslim pupils in the USA reporting that their diet at school consists of tater tots and carrot sticks (17). There have also been concerns raised regarding school dining rooms lacking correct signage where halal food is served, leading to BMSC’s consumption of non-halal-certified food dishes (18, 19). In schools where BMSC are the minority and halal foods are not being served, some Muslim pupils have reported that they are choosing not to eat lunch and are instead returning home hungry (20).

### This study

There are no empirical academic studies that have studied BMSC. Therefore, this study addresses this gap in knowledge. Often, academic researchers focus on school food in primary and secondary schools by measuring objective outcomes, such as dietary analysis of menus and pupil’s dietary intake. However, recently, researchers have increasingly focused on the cultural factors that influence school meal uptake. Currently, there is limited research relating to the provision of religious diets. Past research suggests that a lack of research and understanding on this matter impacts the ease with which pupils maintain a religious diet and has affected how successfully they have been able to benefit from healthy school meal reforms. The limited provision of inclusive food has often led pupils following a religious diet to consume a vegetarian diet while at school, despite this not being their chosen food or preference (4). A gap in academic literature now spanning over a decade highlights the need to address this under-researched topic.

In order to better understand the multiple levels of influence on adolescents’ food choices, Story et al. (21) proposed an ecological framework to consider their eating behaviors’ under four broad levels of influence, including individual (intrapersonal), social environmental (interpersonal), physical environmental, and macro level, to aid in the design of appropriate nutrition interventions targeted at this population. This unique study employs the socio-ecological framework (21) to explore BMSC’s perceptions regarding provision, quality, and choice of halal food in UK schools. This model has four main domains to better understand the perceptions of children, parents, and staff to better support BMSC on school food uptake. The four domains are intrapersonal (individual), interpersonal (social-environmental), physical-environmental, and macro level. The overall aim of this study was to explore the perspectives of BMSC, Muslim parents, and school staff regarding halal food provision in schools and to inform decision-makers on the current perspective.

## Methods

This ARC-funded collaborative study was two-staged. It was developed and co-produced with parents, BMSC, a Northern Children’s third-sector organization, and local authority stakeholders (school food caterers). The city council (DS) and the Children’s third-sector organization (LN) were key partners in this research.

### Qualitative study design

The empirical work was underpinned by a qualitative research design. When conducting qualitative research, an interpretive approach was taken (22). This enabled the research authors to thematically analyze, understand, explain, and interpret social reality through the participant’s reality of the phenomena (23). Data were analysed to look at common patterns to identify factors including barriers and facilitators on food choices and decision-making practices on halal school meals. Braun and Clarke (24) argue that thematic analysis offers the researcher the ability to report the realities of participants, which fit very much with the researcher’s aim to provide an inclusive, descriptive, and detailed account of their thought processes, feelings, meaning constructs, and mental maps. This process not only involves identifying and analyzing patterns (24), but it also interprets hidden and subtle aspects of the concerned phenomena (25). The socio-ecological model was adopted to address the complex nature of the behavior which allowed for deeper data analysis from each stakeholder perspective. This model recognizes that the public health challenges are complex and that decision-makers may benefit from a more comprehensive approach.

A percentile calculation outlined below was conducted to identify schools for participation. Due to schools in three-tier systems (primary school, middle school, and secondary school) not falling into any of the categories calculated, only two-tier systems (primary school and secondary school) were identified. There were a total of 63 primary schools and 10 secondary schools identified. In secondary schools, the uptake of moving to halal options for school meals was 80%, while in primary schools, the uptake was 37%, with two primary schools identified already providing halal food in all school meals being served. In September 2023, Newcastle City Council informed the research team that 21 primary schools had moved to offer halal options to pupils, with non-halal options (meat and vegetarian) also available for pupils at these schools.

### Ethical governance

All ethical protocols were followed in the collection of qualitative data. The project was approved by the Ethics Committee at Northumbria University (Ethics Number: 48003).

Prior to the start of the study, BMSC from community settings (local mosques and community centers) were asked to respond to a short survey via Padlets or WhatsApp about their thoughts on a study for BMSC. This initial approach did not require ethical approval.

When obtaining consent to interview pupils and parents in schools, Loco Parentis’, was initially sought by the schools identified via the selection process outlined below. Schools were supplied with an information sheet about the project as well as the researchers’ contact details. Participants were asked to sign a consent form upon agreeing to take part in interviews that were either face-to-face or by telephone. All data collected in the study were anonymised, with each school and participant being allocated a unique code. These are stored with the signed consent forms in a secure cabinet in a restricted area of the university.

### School selection framework

The local authority representative provided the research team with data on school food provision for BMSC, as well as identifying and evaluating local data on BMSC. Before the school selection process began, data for each school were collated using the following criteria, as shown in the table below (Table 1).

**Table 1:** School selection criteria.

Criteria number	School data criteria	Purpose
1	The religion and ethnicity of pupils attending the school (percentages of BMSC in schools were calculated)	This was to identify how many BMSC attend each school and to ensure that the sample included a cross-section of schools.
2	The schools’ catering management/arrangement system	This was for schools to be inclusive of different catering management systems.
3	Whether halal school meals were already being provided	To understand what the current options were, if any, for those requiring halal food.
4	The school type, i.e., are they part of a trust, academy, or a free school? Are they a primary or secondary school (including tier system)?	To be inclusive of different schools across the primary and secondary school sectors.

A two-step process was followed to select schools to be recruited into the project: (1) Data collected from the percentage of BMSC criterion 1 (as outlined above) in schools were used to categorize schools into those with low, average, and higher than average BMSC populations (seen in Table 2 below). Due to the recognition that the dataset on the percentage of BMSC in schools did not follow the normal bell curve distribution to assign the schools to high, medium, and low BMSC categories, the interquartile range and median scores were used as the statistical measures to calculate the membership boundaries. The interquartile range (IQR) was calculated for both primary and secondary schools, with Q2 and Q3 collapsed to result in three membership categories. It was agreed by the research team to identify two schools from each of these categories that also meet Criteria 2 and 3 (detailed above). All three criteria were used to further select the final schools to ensure robust data were collected. For example, by establishing the school’s catering arrangements and whether these included the provision of halal food. Following this, the six schools in the final selection were then approached to gain consent for participation. Two schools in the low category did not consent, and then the next two schools in that category were selected. This pragmatic approach maximized data collection while respecting the realities of the research process.

**Table 2:** Identified and participating school characteristics.

Primary or secondary school	Percentage of BMSC attending school	Catering system	Availability of halal options	Assigned code
Primary school	High (65%)	Provided by another school	All meals provided are halal	P1
Primary school	Medium (11%)	In-house provider	All meals provided are halal	P2
Primary school	Low (4%)	In-house provider	Vegetarian options only	P3
Primary school	Low (3%)	City caterers	Vegetarian options only	P4
Secondary school	High (35%)	City caterers	Halal options provided	S1
Secondary school	Medium (16%)	City caterers	Halal options provided	S2
Secondary school	Low (3%)	In-house provider	Vegetarian options only	S3

In summary, the utilization of the interquartile range (IQR), data categorization, and participant selection were informed by the unique characteristics of the dataset and practical considerations. These choices were made to conduct a methodologically sound and logical study.

### Participants

Participants who took part in this study via interviews were 15 parents and four school staff members (two school chefs and two school managers). Focus groups were also conducted and consisted of 39 BMSC. Focus group 1 included five BMSC aged 6 to 15; focus group 2 consisted of 12 BMSC aged 12 to 15; eight BMSC aged 11 to 12 were in focus group 3; and 12 BMSC were in focus group 4 (aged 10 to 11). There was also one focus group conducted involving two parents (focus group 5). Three school staff members took part in focus groups, with a focus group involving two school meal providers (focus group 6); and three teaching staff, including a headteacher, took part in another focus group (focus group 7).

All parents and pupils participating identified as Muslim and were from the South Asian community, with ethnic backgrounds of Asian Afghanistan, Irani, Indian, Bangladeshi, African, Arabic, Lebanese, Iraqi, Palestinian, Moroccan, Bulgarian, Portuguese, and Pakistani. Of the 39 BMSC who took part, 17 were attending primary school, and 22 were attending secondary school. Of the parents who took part, 10 participants were parents of BMSC attending primary school, and five participants were parents of BMSC attending secondary school.

BMSC and parents who took part in this study were recruited from schools within the geographical boundaries of Newcastle upon Tyne. The table below outlines the number of participants in the schools selected (Table 3).

**Table 3:** Number of participants in each school.

School code	Number of parents	Number of BMSC	Number of teaching and catering staff	Total number of interviews (including focus group interviews)
P1	6	12	1	19
P2	4	5	0	9
S1	2	12	3	17
S2	3	8	0	11
S3	0	2	2	2
Non-affiliated	0	0	2	2
Total	15	39	8	62

The table below further presents a breakdown of participants from primary and secondary schools (Table 4).

**Table 4:** Total number of study participants.

Participant group	Primary school	Secondary school	Total
BMSC	17	22	39
Parents	10	5	15
Staff (school food caterers)	1	1	2
Staff (school staff)	0	4	4
Staff (non-affiliated)	0	0	2
Total participants	28	32	62

Two parents who consented to take part in the study withdrew consent to be audio-recorded but consented to handwritten notes being taken by the researcher. Two interviews were conducted by telephone at the participant’s request.

There was a reduced number of school catering staff participating in this study due to unanticipated governance barriers encountered during recruiting. Some field work coincided with Islamic fasting periods; therefore, a small number of interviews were rescheduled to be conducted post-Ramadan.

As mentioned earlier, a pre-requisite of this study was to include Public Involvement and Community Engagement (PICE). The PICE informed the direction of the research design from the inception of the project. Both parents and BMSC discussed and piloted how the participants would respond to the research. All public participants were reimbursed for their time.

A group of BMSC (x7) were recruited from a local third-sector organization that specializes in teaching Arabic to Muslim children. In addition, a small group of parents (x5) from the community were also recruited. All BMSC and parents said this was an important area to explore. BMSC confirmed that they felt comfortable about sharing their views on school food. Most of these parents and pupils formed part of the community engagement team, referred to as Public Engagement and Community Engagement (PICE), and their involvement spanned the development, conducting, analysis, and dissemination of the project. It was felt that the strong community connections with the research team and PICE members were an important factor in this continuous and consistent involvement of parents and BMSC.

### Qualitative data collection and analysis

Data collection was time-sensitive, and the majority of data were collected during term time by conducting semi-structured and focus group interviews. Interviews focused on exploring participants’ experiences of school meals. Relevant individual demographic data, including age, gender, and ethnicity, were also collected for contextual purposes.

Interviews were digitally recorded and transcribed verbatim. Identifiable information was removed at transcription. To ensure rigor, transcripts were subject to the research team and public participants (×3), who scanned transcripts and identified general statements and themes from the data (26). To facilitate thematic analysis, the research team used the software package NVivo (V.12). Three themes and their sub-themes were identified and are presented further down in this article.

### Public involvement and community engagement (PICE)

There were three PICE meetings in total. The first meeting involved an initial meeting at primary school gates with parents who were known to the research team. It also involved approaching parents in local mosques and community centers by attending social gatherings and other family events. As time progressed, parents confirmed they were learning more about their advisory roles. Parents felt that they could not find a school that was both seen as highly academic and that provided adequate catering for their children’s religious needs. The parents advised that qualitative research interviews and the topic guide questions were appropriate, and the vocabulary used for the questions was understandable and could be used for data collection.

The second PICE meeting involved discussing recruitment for the study and what taking part in interviews and focus groups would include. The third PICE meeting, following the beginning of the interviews being conducted, involved discussing the interim findings and asking PICE participants for their feedback regarding themes they had observed within the transcripts. This was done via video call due to the meeting coinciding with Ramadan. During this meeting, parents reported that the results found in the anonymised transcripts were as they expected when they were presented. It was fortunate that committed members took ownership of this group and tried to attend all sessions and make valuable contributions.

## Results

This section reports three themes from the thematically analysed data collected via interviews with BMSC, parents, and staff. Thematic analysis led to three key themes being identified regarding the perceptions of BMSC and the extent to which BMSC and their parents are able to contribute to school food decision-making in their school. These themes were: provision of halal food, referring to the availability of halal food provided in schools for BMSC and the variety of options available for children following a halal diet; BMSC inclusion, referring to the extent to which there was ease and provision for BMSC to feel able to practice their religion in the school dining hall; and school food decision-making, exploring what expectations of inclusion for BMSC to meet their religious needs were catered for in primary and secondary school. The socio-ecological framework allowed the research team to order the data within its respective domains (21) with the view of understanding from a theoretical underpinning the complexity of the facilitators and barriers of BMSC and the uptake of school food.

### Theme 1: halal school food provision

#### Sub-theme: provision of halal food/availability

All BMSC interviewed demonstrated an understanding regarding what halal food is and how it should be prepared to some degree:

*If you eat an animal, it needs to be killed with one of the names of Allah.* (P1, BMSC, Focus Group).

Most primary school BMSC reported feeling included in their school meals, with them being able to consume both halal meat and vegetarian options. However, at the *interpersonal* level, this empirical study highlighted that some children who were unable to eat the school food on offer in the dining hall said they would, ‘eat whatever’, when they were ‘really hungry’. The quote below illustrates that BMSC consumed school food to stop feeling hungry:

*It’s just easier to like choose foods and, so you are not hungry anymore—So you are not like hungry when you go home.* (P2, BMSC, Focus Group).

A BMSC who was lactose intolerant said that he would then, ‘deal with the consequences later’ (S1, BMSC, Focus Group). Another child said that the school introduced chicken burgers but that there was not enough for everyone, and on occasion they would run out before being served, leaving their options limited to ham sandwiches (S1 BMSC Focus Group 1). Parents of children from this school felt it was better for schools to opt for halal food as non-Muslims could eat all the options available, whereas if the food is not halal, then some pupils were restricted with their choices (S1 Parent 1). Parents felt the school food options were inclusive, but they also reinforced that it was a privilege when halal food was provided. The parent below stated that for a school to have a variety of school food on offer at lunchtimes demonstrates a level of inclusion for BMSC:

*To have that variety, that option available, it makes them feel included*. (P2, Parent, Focus Group).

Repeatedly, parents of BMSC stated that each child was different and that they were aware that some were fussier than others when eating food:

*Just my oldest son is little bit fussy at home also so, I’m not sure if he’s eating well or not, but the second one is eating, he likes anything*. (P2 Parent 5, 1:1 Interview).

The catering staff above explained how they need to assess if addressing the needs of a child’s dietary requirements would be economically viable for them in schools. Sustainability and increased costs to provide specific meals to meet children’s dietary requirements were a challenge for schools:

*I know it’s a horrible thing to say but it’s one of those things that you have got to think for one child with a medical condition, so it’s got to be halal and that child obviously might eat 5 days a week, might be packed lunch 2 days a week, it depends on how much they are going to cost. But you could be talking £5,000 outlay for a new bench, pots, pans, whole area and things like that for maybe something that you are going maybe get £1,500 in return for*. (P1, School Food Caterer, 1:1 Interview).

From a theoretical perspective, at a *social level*, some parents said they were more concerned about their children’s general eating habits. In this current empirical work, it was explained that their children were ‘fussy eaters’, and it was difficult to get in touch with the school to discuss their children’s dietary requirements and explain what they could eat. In contrast to the parents’ views, some of the BMSC at these secondary schools stated that they provided menus online, and children at both primary and secondary schools said they could ask the catering staff about the school foods being served at lunchtimes.

There were different experiences on halal food consumption expressed by BMSC from primary and secondary schools:

*I think a lot of schools can have Halal because they all can eat, whereas other meat they use, Muslim people cannot eat, so it would be the best option that they did Halal*. (S1, Parent, 1:1 Interview).

The school staff and catering staff stated that they had met with parents regularly, and some staff would even provide them with recipes and tips on the food they cooked, but more importantly, they said that the communication between parents was strong. The quote below is from a primary school staff member:

*So, the parents always know what’s on the menu. I’ve never had anything bad come back from parents*. (P1, BMSC, School Food Caterer).

At the physical level, some secondary school children stated that there were halal meals on offer, but by the time their turn came to be served, there were no halal options left. It was felt that this was because some children who did not have a halal food requirement would also select the halal option:

*I tried having the Halal option but with the number of Muslim kids, it was not able like—and not everyone would get it*. (S1, BMSC, Focus Group).

Some children said it would be helpful to introduce labeling and packaging for them to know which food was halal. They commented further on this, stating that it would be helpful for this signage to be written in Arabic. Responses from primary and secondary schools about the provision of halal food were mixed and related to the percentage number of Muslim children in their schools. For example, a chef in a primary school used a business model to explain the cost-effectiveness of providing halal food to a pupil: ‘it depends on how many it was for stated’ (P1, BMSC, 1:1 Interview). Primary school staff and children were positive about the fact that children could taste new foods being served and felt that they were at an advantage, especially if it was a teacher encouraging the children to taste different foods at lunchtimes:

*Because we are nursery to Year 6, so it’s still a case of, but they will try it if the teacher is, say oh are you having this, can I try a bit?* (P1, School Food Caterer, 1:1 Interview).

On the other hand, pupils interviewed from the secondary schools said that they felt a level of exclusion. This was because they said they watched non-Muslim pupils eating warm lunches/meals such as lasagne and shepherd’s pie, with them unable to opt for these school meals because the meat was not, ‘*halal*’, but ‘*haram*’. while there was food transparency in the schools and school food menus were available to the children, it was highlighted that the limited options available to BMSC, such as chicken and Quorn, were not reflective of their food choices, particularly their food preferences at home. It was also stated by a secondary school parent that they felt ensuring their children consumed culturally inclusive, healthy, and nutritious food at school and home was a constant challenge. A few parents said that menus should reflect what BMSC consumes at home:

*They should keep in mind what the children eat these days you know what they have on their menu is yes kids eat chips*. (S2, Parent, 1:1 Interview).

The parent in the next quote said that she tried to make healthier home food options:

*My kids they are not like vegetable kind of people they do not like vegetables and that’s why when I’m cooking at home if I’m making a lasagne I put vegetables in there and if I’m making a shepherd’s pie, I put vegetables in that so that they are still getting their vegetables*. (S2, Parent, 1:1 Interview).

There were BMSC interviewees who knew the name of the food caterers and were kept informed of changes made to their catering. Interviews with the primary school food caterers revealed that the staff were aware of the days some children would bring in a packed lunch, in this case for 2 days, Mondays and Tuesdays, and have school dinners on Wednesdays, Thursdays, and Fridays because they liked the pizzas on these last three weekdays. At the *macro* level, a parent who had worked for schools in food catering explained in detail how she managed to change the school food purchasing policy to local halal food from HMC-certified butchers only. School staff, where there was a higher percentage of BMSC, were positive about their approach in addressing the diversity of the school and the provision of halal school meals:

*I think the positive is that we are acknowledging the students that we are a diverse school*. (S1, School Staff, Focus Group).

Schools with a low percentage of BMSC did not consent to take part in this study. It was noted that the perceptions were different for staff members employed at schools where halal food was not being provided and there was a low percentage of BMSC in attendance compared to the perceptions of staff from schools with a higher Muslim child percentage.

Secondary school staff reported an awareness of how BMSC were having to make decisions in the dining hall that non-Muslim pupils do not, with non-Muslim pupils being able to decide when they will have a hot meal based on preference, whereas BMSC’s decisions often include opting out due to the food being haram in the empirical findings:

*Interviewee 1: No Muslim students would go home hungry, because it’s always halal food options. But you do get some, you do get some kids who are who are white English Christian who might not like the food options and just say, I do not fancy the hot food today. I’m going to go hungry. But actually, we have not heard of anyone.*

*Interviewee 3: No, I’d agree, you are absolutely right, there’d be some children who would choose not to have, but not because there is not an option for them, do you know what I mean?*

*Interviewee 2: But there’s always a range of sandwiches and wraps, pasta.*

*Interviewee 3*: *So you know, guests have heard of children not selecting anything, but not because of their beliefs. So they have not chose something because it simply wasn’t something that they wanted*. (S1, School Staff, Focus Group).

Staff were aware of the school food choices that both primary and secondary schools could offer:

*Primary schools, that are fully halal. They do not offer anything other than a halal meat option… In secondary schools there’s an option*. (Not affiliated to school, School Food Caterer, Focus Group).

Suggestions about improving school food ranged from BMSC from both primary and secondary schools on increasing halal school food choices to more time needed for lunches:

*With the growing Muslim children, I think there should be*. [more halal food on menu] (S1, BMSC, Focus Group).

There were many suggestions on improvements to school food from parents including what their children consumed at school, or the lack of. As the research team further probed these responses, it appeared that this was linked to access to information and schools on wanting to speak to teachers/school catering staff to verbally discuss their concerns or their children’s requirements.

*They eat like the burger and sorry, like sandwiches and he says we eat sometimes chicken and he did not mention any vegetables. I’m not sure maybe he not choosing or something like this*. (P1, Parent, 1:1 Interview).

### Theme 2: BMSC dietary requirement inclusion

Many children felt that they were expected to eat the vegetarian option if there were no halal food options available. Some children stated that this was the norm for them. There were parents who felt that the food options for their children could be more nutritious, and thought the portion sizes were inadequate:

*I expect them to have a nutritious meal and I do not believe that they are receiving a nutritious meal and the reason I know this is because I’ve had meetings with the school not for my younger child it’s more the older but that’s to do with dietary requirements anyway but I know from the portions the way they give them out and the way they are offered it’s just like there’s not enough I do not believe there’s enough* var*iety I really do not not with school lunches*. (S2, Parent, 1:1 Interview).

The parent above stated that she had been in the school, and there were a number of aspects that could be improved, including food quality and choices provided by schools. One child in a secondary school said that the halal school food provision was limited. In the quote below, it can be seen from the empirical work that the child felt there was only one halal food option, halal chicken sandwiches:

*They tried to introduce Halal food but it was just like a chicken sandwich*. (S1, BMSC, Focus Group).

However, the school staff in the same school explained how there were several food choices available for BMSC:

*Daily food choices’ there’s four choices on every day in a primary school, and at secondary school it can be anything from seven up to 11 choices*. (Not affiliated to school, School Food Caterer, Focus Group).

Primary school staff explained how they encouraged children to taste foods on display and verbally explained the appeal of the foods being served to encourage tasting unfamiliar foods.

Many children said that they did not want to ask the school staff if the food was halal or haram. Parents suggested better signage of foods so the children could read what foods were halal and said this would facilitate the access to school meals in the dining hall.

Moving from this *interpersonal* level, the empirical findings highlight that the parents said that the level of anxiety and worry may also be linked at a *social* level and involve stigma, especially in secondary schools when the children had to ask the school catering staff about which dishes were halal.

*My younger child is she’s really reserved and she says she’s sure it’s halal and I say can you not assume you need to ask. They feel a bit intimidated to ask may be because they are children and they feel a bit shy and a bit nervous. So I think it would be better for the children for everything to be labelled*. (S2, Parent, 1:1 Interview).

The parent above explained how her child would opt for the vegetarian option, as this was more comfortable for her child. On the other hand, in a primary school, one parent felt the school meals provided were nutritious and nutritionally compliant:

*Nutrition wise, there’s plenty there. They’re getting a bit of everything. Plenty of sugars, plenty of fats, plenty of protein, and the portion size is good as well. I mean xxx, he does come home and say that he does not finish his lunch.* (P1, Parent, 1:1 Interview).

A general concern raised by parents centered around the halal food consumption, where young BMSC forgets to ask whether food was halal, there was no provision within the school preventing them from eating non-halal food:

*It could be chicken and they can eat it, but they cannot it’s not halal. It needs to be labelled and that’s why they have to repeatedly ask*. (S2, Parent, 1:1 Interview).

There were mixed views about portion sizes among parents. Staff at both primary and secondary schools stated that they have meat and non-meat options, *jacket potato fillings, there’s soups, there’s, you know, there is Quorn products on, obviously because we use that as some of the proteins, there’s pizza, there’s fish, so four choices on every day* (Not affiliated to school, School Food Caterer, Focus Group).

At the *social* level, children in secondary schools said they missed the decision-making systems in primary schools they had for eating meals:

*Because in my primary school we had like a voting system and like, it was very much—everyone made a decision and we used to have like, Halal chicken burgers with like, a side and dessert and it was all—So a meal, an actual meal*. (S1, BMSC, Focus Group).

Parents with children in secondary schools felt that the supervision for BMSC as they grew older was limited. The quote below was from a parent about her child in a primary school and how she felt the supervision during lunchtimes for her child changed as the child moved classes:

*I do not think they check as they get older. I mean, I think in key stage one they do check, but now that he’s in key stage two they do not. So, they’ll tell him off for having ate all your food, and he’ll just say that he does not like it, or he just does not want it. And then I would say because all they do is they’ll chuck that food in the bin. But if there’s a brand new, say for example, an apple that he has not ate, put it back*. (P1, Parent, 1:1 Interview).

The palatability of the food was an issue for some children, and some children said they would eat outside of school for lunch because the food was cooked in a Western style and lacked spices. The secondary school children also stated that sometimes they would have no other options and eat crisps for lunch:

*They would make like curry, but you know how it’s like? Oh, like I’m not trying to be racist but, you know, like the white version of a curry? Without any other spices and all of that*. (S1, BMSC, Focus Group).

Some children did feel that the halal curries that they were being served at school were different from the curries they consumed at home.

One child at a secondary school said he felt more tired in the afternoons, and this was because he would only have crisps for lunch. He said that the school dinner queues were very long, and that this was a key contributory factor that led to poor decision-making choices in secondary schools. The following parent felt the school food in the primary school her son attended was sufficient, and she was content with the school meal provision:

*Nutrition wise, they have a little drink, they have a full meal, they have a little dessert. I think it’s definitely enough to fill them up whilst they are at school, from morning to obviously the end of school, it’s definitely more than enough*. (P1, Parent, 1:1 Interview).

The school food catering staff expressed how knowledge exchange was an important aspect of their role. One staff participant said that they always made an effort to understand what foods the children would like to eat and would make time to listen to parents about how to include the correct spices in meals. Learning recipes from parents was an approach some food caterers used, which was positively perceived by the parents and BMSC. It was clear that more learning on halal food by staff was recognized by some of the participants:

*A lot of staff who have been around for 40 years and I think there was always a fear that we could not serve a halal, it was the kitchen has to be blessed, you have to have separate pans. It would be educating people around, you know, it’s not as difficult as you think it’s going to be*. (Not affiliated to school, School Food Caterer, Focus Group).

Catering staff from primary and secondary schools said that they always listened to parent concerns on school food and where possible, they would take action on parent requests. One staff member from a secondary school stated that they ensured that they were up to date with the school food standards and would ensure school food would comply:

*Whenever there’s a change in legislation, or a change in the menus that we produce, so when we need to train staff up, that’s standard, we have got that as well. To my head, think about the vegetarian option, you know, you are not going to cross contaminate that with the meat product, so it’ll be, so there’d be a system in place, this is how you would have to produce your meals and your tins would be these tins and this would be this. So, I think there was a bit of a fear, there, around what if we did, they do not only with the halal food, but we also get it now with, they are terrified in case they give a child who should not eat meat, the meat burger. So, it’s that, it’s that worry. We want a bit of education around, you know, it’s not so difficult to do, it’s just about understanding*. (Not affiliated to school, School Food Caterer, Focus Group).

Training was an important aspect for the school above because there were some fears around the provision of halal school food.

Schools, on the other hand, were making changes to the school food menus to address the halal food requirements of their pupils. These menu changes involved substituting halal meat where non-halal meat was used to cook the meals, e.g., chicken korma.

At the *physical* level, there were some children who felt that there were limited options for them:

*Like there’s some days there’s not much options really*. (S3, BMSC, Focus Group).

The school kitchen sizes and facilities where barriers were also identified by staff at the *physical* level as well as cross-contamination factors, ‘especially new schools because the kitchens are the tiniest places, now, in the school and we do not have a lot of storage’ (Not affiliated to school, School Food Caterer, Focus Group). Schools, at the *macro* level, stated they were making progress with providing halal food and variety of these foods, but there remained issues with pupils with special dietary requirements, e.g., lactose-intolerant BMSC, a dairy intolerant BMSC felt there was very little choice for him at school:

*They tried to introduce Halal food, but it was just like a chicken sandwich, and it was like, oh, if you have dietary requirements, just have the vegetarian meal*. (S1, BMSC, Focus Group).

Some parents resonated with this statement and said there were not a lot of options for their kids to choose their meals from, ‘I feel like when they come home and they say to us like—you know, “I could not have any food today because there’s not more options for me to eat it”’ (S1, Parent, 1:1 Interview). Home-cooked food had hidden vegetables, ‘I’ll make chicken pasties and they are really intrigued because I really finely cut carrots, peppers’, describing recipes and willing to share their methods with schools (S1, Parent, 1:1 Interview).

Two primary schools in the locality were fully halal, but catering staff said that the secondary schools have options with meal deal purchases, and halal food was an option.

Overall, at the macro level, the main concern parents had was that their children should be consuming halal food at school, and it was a worry for them if this was not the case:

*And I do not think everything is clearly labelled either because I tell my son make sure you ask if it’s halal and do not assume because everything is not halal and children forget to ask and we are conscious, but children aren’t. I mean they might be getting fed something and they have not asked they have assumed that it could be chicken and they can eat it, but they cannot it’s not halal. It needs to be labelled and that’s why they have to repeatedly ask*. (S2, Parent, 1:1 Interview).

Staff were continuously looking at ways to introduce a variety of school foods for the pupils. It can be seen from the quote below that the school staff and catering teams were looking for options to provide wider options:

*Say there’s a chicken korma on, on the menu currently at the minute it’s a chicken korma, or the alternative is a Quorn korma, so it’s trying to sort of looking at putting a menu together so that they could have the chicken korma, but they could have a halal chicken korma. Or, introducing, when the sausage is on, introducing a chicken sausage that’s a halal sausage. It’s those, it’s trying to adapt the menu that we have currently got, where we can bring in these products. So, that’s going to, so we have agreed, I mean I have not done the menu yet, but I’m in the process of doing the* menu. (Not affiliated to school, School Food Caterer, Focus Group).

BMSC did find the school food being served in schools appealing, but sometimes they were unable to choose these foods because they were not halal:

*It’s like sometimes I’m like craving something, like I want to get like a burger but it’s like pork so I cannot get it but I’m craving it. Yes, I just want that to be like halal so it’s easier*. (S1, Parent, 1:1 Interview).

### Theme 3: school food decision-making

Children were very vocal about the food they consumed at school and stated that they wished there were more variety and choice. Decisions on selecting school food were based on the better option at both primary and secondary schools. The empirical findings showed that at the *interpersonal* level, some children said that they would like to eat a healthy, nutritious meal, but this was not always possible.

There were some children who said that they would go without food all day at school or eat only crisps because they felt the food was not palatable:

*You would either have to skip lunch or have a crisp, or like a juice*. (Not affiliated to school, School Food Caterer, Focus Group).

Secondary school children said that sometimes there was a lack of appropriate school food and that they had to settle for non-nutritious food.

The parents of these children did feel that it was better that they had something rather than nothing; ‘if he eats even a little bit, he will not be as hungry as if a child goes early in the morning into the school and comes back at three o’clock and he has not eaten anything filling’ (P1, Parent 1:1 Interview). However, parents were forthcoming in their narratives about the assurance from schools that their children were eating food that was permissible in their religion:

*At least we know that he’s not eating something which is not permissible in our faith*. (P1, Parent, 1:1 Interview).

Food catering staff, on the other hand, worked hard on developing a 3-week menu cycle and tried to vary foods where possible, e.g., potatoes, chips, and mash. There were also attempts from school food caterers to completely change, ‘over to a halal meat menu, which was quite restrictive because it was just lamb and chicken every day’, but the school meal uptake did not increase and so only ran for a year as it was also expensive to run and it was quite restrictive with chicken and lamb every day. School staff from secondary schools were proud of their school meals for meeting the needs of their diverse pupils. Some secondary BMSC were unable to finish their meals because it was too bland for them and said that it, ‘ends up in the bin’ (S1, Children, Focus Group).

Staff, both school staff and catering staff, were very positive about their variety of food choices but also very proud of their multi-cultural approach to provision of school lunches:

*So basically, on a three-week menu cycle, I think they have got mashed potato 1 day, new potatoes on 1 day, and I do a baby, what I call a baby jacket potato, so basically, it’s a new potato boiled and then baked in the oven so, like little mini jacket potatoes*. (Not affiliated to school, School Food Caterer, Focus Group).

*We’re really proud that our school is multicultural, we are complying with all the school food plan … the burger is probably the most popular thing in the school. So as many children as you can imagine taking it but not because it’s a halal option, just because it’s a burger*. (S1, Staff, Focus Group).

At this *social* level, it was reported that ketchup would be purchased as a side dish because the food was dry and not palatable for them. Due to a lack of choice, children said that they reluctantly had to select food that was halal, but it was bland, and they bought sauces to add for taste:

*You have to buy ketchup on the side because it’s really like, dry and not like nice*. (S1, BMSC, Focus Group).

There were some parents who said that they empathized with their children if they were hungry at school and could not concentrate:

*And that makes them tired and drained and not really concentrating and I would not be able to concentrate anyway if I’d not eaten much and you know your stomach’s rumbling and you cannot really concentrate in school can you*. (S2, Parent, 1:1 Interview).

Parents said they were unsure of what to say to their children when they would come home, and their children did not eat the food because they did not like it; hence, they felt this was a negative aspect of school meals, ‘this is something which is not in our control’ (P1, Parent, 1:1 Interview). In secondary schools, some children felt that the queuing for dinners left them with little time to eat their food. A few children said they had to throw their food in the bin because there was not enough time left for them to eat their lunch. There were other parents who were ‘topping’ up their children’s accounts, but they were purchasing a lot of unhealthy sweet foods such as waffles and desserts. One parent was concerned that her child was selling sweet foods and buying other foods after school:

*When I look at what he’s purchasing, it’s a lot of waffles, desserts, extra drinks, cookies. And he just seems to be selling them to get that money and then he just spends that after school*. (S1, Parent, 1:1 Interview).

From the *physical* aspect, the long queues were perceived as barriers to eating in school at lunchtimes:

*You have to go all the way around, outside, all the way to the canteen and be in the line for ages, and then you get to go in, and by the time one of the—you only have 5 minutes eating you have to go into a lesson*. (S1, BMSC, Focus Group).

Some parents felt that the consumption of fast foods at schools destroyed their taste buds:

*Because like fast foods like destroy their taste, the burgers, pizzas and these thing’s*. (P1, Parent, 1:1 Interview).

A parent said she was upset as she would transfer money to her son’s account and her son would only eat chips as there was not a lot of option for him. This was an issue for staff who were trying to sustain costs as prices were going up for foodstuffs. The school food caterers were exploring ways to keep prices low but also increase school food uptake with less food wastage. For example, the school food caterers were aware of introducing a variety of more halal school meal choices, ‘I’d love to be able to put a beef dish on but once again my butcher will not supply me with halal beef it’s too expensive’ (P1, School Food Caterer, 1:1 Interview).

At the *macro* level, children said that they would prefer their food displayed with labels so they knew which food was halal and better halal food presentation, and they would enjoy their food more:

*I think it would make us more enjoying of school*. (S1, BMSC, Focus Group).

When parents were asked about the importance of their children eating halal food, based on the analysis of the empirical data, most said this was very important:

*I mean if you follow your religion then it would be important. I mean I follow Islam, so it’s very important to me. It’s very important for my son to eat halal*. (P1, Parent, 1:1 Interview).

While, many school food caterers ensured that schools had options, some schools where all food was halal had less to consider when making halal meals, ‘*Because it’s the whole picture for me, it’s not as if I have to worry about having a separate bench to do halal, having a separate area in the kitchen to do other stuff*’ (School Staff).

Managing a general increase in price was a major concern for the schools interviewed. School staff and catering staff were aware that the cost of meals for children should be kept as low as possible:

*We’re desperately trying to not increase food prices and keep them the same, the cost of the meals for the children, so cost is a massive issue*. (Not affiliated to school, School Food Caterer, Focus Group).

The set-up the school had with the provision of school meals was an important factor in the food choices and variety, as well as cost. In the school below, the school food caterer stated that they sent out menus to their school group, who had the opportunity to have input into the menus:

*We set the menu and then the menus are sent out to each head teacher or business manager who may well be in that school and then they can have some input into what’s on the menu and any changes that need to be made*. (P1, School Food Caterer, 1:1 Interview).

## Discussion

The aim of this study was to explore the perceptions of BMSC, parents, and catering and school staff regarding the provision of halal food as part of school meals. This research was co-produced with children and adult PICE groups from the inception to the dissemination of the project, and regular meetings with the PICE groups were conducted to strengthen knowledge mobilization and support engagement across all stages of the project. Thematic data analysis identified the following three key themes at school dinners: Halal school food provision refers to the availability of halal food provided in schools for BMSC and the variety of options available for children following a halal diet; BMSC dietary requirement inclusion refers to the extent to which there was understanding across stakeholders on provision for BMSC and to feel able to practice their religion in the school dining hall; and school food decision-making explores what expectations of inclusion for BMSC to meet their religious needs were catered for in primary and secondary school. The key findings illustrate that schools catered for diverse pupils’ food requirements, including BMSC dietary needs; however, food choices were limited. It was perceived that there was a lack of choice and/or insufficient food. In some cases, it was reported that the BMSC would not eat at school and wait until they were home. BMSC and parents would like to be more involved in the decision-making process; although some were involved, the impact of this involvement is not clear. Staff—both catering and school staff—felt that cost implications contributed to their menu development process, but they do attempt to make food to meet the pupils’ taste preferences, and there was a willingness to learn about the cultural food recipes. Academic research in this area is sparse. Interestingly, there are increasing reports by local city councils and third-sector religious organizations generally under the equality agenda for schools to provide halal food. Lancashire City Council has developed an Equality Analysis Toolkit for decision-makers on the supply of halal meat to schools (27). Further work in this area is suggested to build on the knowledge obtained in this current study, in particular evaluations to understand local school initiatives on the provision of halal food in the absence of a statutory requirement to do so.

Globally, the 2005 Convention on the Protection and Promotion of the Diversity of Cultural Expressions became a legal instrument with seven objectives, including ‘to protect and promote the diversity of cultural expressions.’ Importantly, schools were recognized as important platforms to transmit information and knowledge on protecting these expressions to young people, and resources were made available to translate to local contexts. Within the current study, at the interpersonal level, all BMSC demonstrated an understanding regarding what halal food was and how it should be prepared. BBMSC, parents, and school staff all recognized the importance of offering halal food choices at school. Local council staff and school staff were evidently receptive to engaging in knowledge exchanges, in line with the policy: ‘to give recognition to the distinctive nature of cultural activities, goods and services as vehicles of identity, values and meaning’ (28). However, all schools approached (at least six) in the low categories declined to take part in this study. This study highlights the need to further understand how provision, quality, and choice are perceived in low-category schools.

This study identified key barriers to the provision of culturally appropriate halal food for these school pupils and how these have manifested into a range of positive and negative aspects of the provision of school meals. The UK government policy has driven the provision and delivery of school meals, and the School Food Standards were introduced with a view to preventing nutrient deficiencies initially (29, 30). When collecting information on school food provision for BMSC, it became evident that schools where halal meat was not being provided on a regular basis left BMSC with limited options to choose from in the school dining hall. This created an unwanted restriction on the food choices BMSC were able to eat, resulting in fewer food choices being made less on preference and more on what was available that was in accord with their religious requirements.

The aim of the school food policy is to create healthy subjects by providing space to learn and thrive (31). Healthy school meals can encourage long-term healthy food behaviors in pupils, and the school environment is viewed as a strong platform to promote healthy eating behaviors due to the amount of time spent in school (32). At the macro level, the empirical results found that school food caterers and staff were aware of their diverse pupil groups and were keen to develop their menus to be inclusive. School and catering staff members explained in detail the efforts they had made to include BMSC in the dining hall, with BMSC in high categorized schools corroborating this by stating that they had been given the opportunity to be included in school food decision-making via school councils and committees. These opportunities for school food decision-making fall within the intrapersonal domain of the sociological model, and empirical data illustrated that children and parents were being consulted on school food choices. It is, however, worth noting that the staff who agreed to be interviewed were from schools where halal food was already being provided for BMSC, with a high percentage of BMSC in attendance in these schools.

The practical implications in the dinner hall were linked to availability and variety. The halal food was sometimes not available for pupils who had entered the school dinner hall late. More variety of halal food was suggested as children said that they wanted to eat something different, e.g., they would have chicken burgers every day. It is clear that staff encourage BMSC on school meal engagement, but it is not as clear as to how to obtain a balance between school food meal provision and variety.

A healthy diet is recognized as having a positive impact on children’s mental, physical, and emotional wellbeing (33). Overall, the findings in this study demonstrated that where schools are providing halal food, there are often limited options for BMSC, with choosing a school meal becoming less of a preference and more based on maintaining religious requirements. BMSC were unable to choose between meals based on preference in comparison to non-Muslim pupils. This study suggests further research to understand how such barriers can be managed.

The findings from this research were consistent with previous literature, where it had been reported that BMSC were often limited to vegetarian options (4), which was highlighted at interviews. Both schools and school food caterers were keen to seek further knowledge regarding BMSC and food provision; therefore, further work is suggested with these stakeholders to understand their perspectives on uptake, especially in terms of designing 3-week menus inclusive of BMSC that are imaginative, flexible, and nutritious. This is in light of the argument that catering managers may be instrumental in effective guidance from the government on School Food Standards, e.g., discrete vegetable inclusion, convenient menu options, and combinations of different foods on menus (34–36). American literature reports that Muslim pupils are not eating lunch and instead return home hungry (17, 20). This was corroborated by BMSC in this study, where BMSC stated that often they will return home hungry after choosing to opt for snacks over the vegetarian option available. Attempts by parents in this study to hide vegetables in food to encourage their children to consume healthier food were also explored in secondary schools with adolescents; however, although quality was an influencing factor, peer pressure played an instrumental role, and catering staff stated that the ‘grab and go’ options were more popular than sitting at the table with knives and forks (36). It is argued that as pupil diversity increases, school food caterers may want to consider such tactics as hiding vegetables in school food because clearly visual appeal, familiarity, time/convenience, and peer influences play an important role in school food choices (35). However, the balancing of inclusivity from a cultural and religious dietary requirement of BMSC against the current period of austerity and rising costs are challenges. Hence, there were multifactorial factors that BMSC in this study felt hindered them when it came to school food uptake.

This study also recognizes the intergenerational implications that parents and their children may experience during mealtimes at home and school. This complexity could be a factor overspilling into school meals, hence the lack of school meal uptake. The empirical findings suggest that take-up is low mainly due to the halal food choices and timing on entering the canteen, and further work is suggested to understand the influence of practical implications in the dinner hall and barriers identified. The range of cultural backgrounds and meeting global food preferences maybe a challenge, but meeting local demographic school food requirements, as some schools were adopting in this study, was encouraging in addressing diverse pupils’ religious requirements. Children in this empirical study who were medically intolerant to certain foods said that they had to suffer ill-health after consuming foods they were intolerant to because there were no other appropriate options for them. However, more in-depth work is suggested on what BMSC with food intolerances perceptions are of school food choices. Preventing hunger has a social justice implication. This study is unique in its findings and provides insights into the perceptions of key stakeholders about halal school food in a northern city of England. This study should support decision-makers on the provision of school food for BMSC. In particular, where the recommendations may be simpler to adopt, e.g., serving English meals with halal meat, there may be signage to reduce stigma in the school dinner hall. Kracht et al. (37) suggested interventions, to include teachers and the curriculum, to promote healthy weight-related behaviors in priority populations. The food environment plays a vital role in school meal consumption (38). However, this empirical study reveals that some of the barriers are general to the wider pupil population (39). For example, time pressure may negatively influence the school meal experience for children when the dining halls are overcrowded, especially as found in this study that there are specified time slots for lunches and the BMSC and parents interviewed said that they prefer to go hungry at school and wait until they get home to eat.

There are a number of limitations in this study, such as the scope of the study, which is a small qualitative piece of work. It is also recognized that the economic climate for local councils has been a struggle, and this may have impacted further developments in the provision of halal school food. Nonetheless, the findings may be informative for other schools with BMSC across the UK. The schools that took part in the study were in either the medium or high category; therefore, it is suggested that schools with a low percentage of BMSC should be studied, in particular, to explore the perceptions of BMSC attending the low-category schools identified. There is also a low number of school staff and school food caterers compared to BMSC and parents interviewed in this study. It is suggested that school staff and school food caterers could provide a deeper insight into the dining hall setup and food-eating behaviors of BMSC. It is also insightful to learn that during religious festive periods such as Ramadan, project timelines could be setbacks, and hence it is useful to align empirical work with the Islamic calendar. This is because some BMSC fast in school, with this lasting for a whole month; therefore, interview responses could be watered down as they do not consume food at school during this time. Further work is suggested on exploring stigmatization of halal school food consumption in primary and secondary schools. But also, how to engage secondary school BMSC in the uptake of halal school food and for school food caterers to prepare the food so it appeals to the BMSC rather than providing food where taste is lacking.

In conclusion, this study employed the socio-ecological framework (21) and contributed to knowledge of BMSC’s perceptions regarding provision, quality, and choice of halal food in UK schools. It was evident that the dietary needs across primary and secondary schools in Newcastle upon Tyne, where the percentage of BMSC in attendance was mid to high. This study illustrates how essential it is to provide halal school food for BMSC that fosters an inclusive and respectful educational environment. The attempts by the school staff and school food caterers to involve children and parents in decisions and planning were perceived to raise awareness and support to incorporate religious diets into the School Food Standards. This involved collaborating with local halal meat suppliers that helped to overcome challenges in halal meat provision. Schools ensured that BMSC had access to culturally appropriate and religiously compliant school meals. There is further work, however, required in the dining hall to enhance palatability, address practical considerations such as variety and taste, and address religious requirements as diversity continues to increase in British schools.

## Data availability statement

The raw data supporting the conclusions of this article will be made available by the authors, without undue reservation.

## Ethics statement

The studies involving humans were approved by Northumbria University ethics committee. The studies were conducted in accordance with the local legislation and institutional requirements. Written informed consent for participation in this study was provided by the participants' legal guardians/next of kin.

## Author contributions

ZS: Conceptualization, Data curation, Formal analysis, Funding acquisition, Investigation, Methodology, Project administration, Resources, Supervision, Validation, Writing – original draft, Writing – review & editing. SC: Supervision, Writing – review & editing. LL: Writing – review & editing. MAD: Supervision, Writing – review & editing.

## References

[1] Office for National Statistics. (2023). How life has changed in Newcastle upon Tyne: census 2021. Available at: https://www.ons.gov.uk/visualisations/censusareachanges/E08000021/

[2] United Nations. (2024). International migration. Available at: https://www.un.org/en/global-issues/migration

[3] Office for National Statistics. (2022). International migration, England and Wales: census 2021. Available at: https://www.ons.gov.uk/peoplepopulationandcommunity/populationandmigration/internationalmigration/bulletins/internationalmigrationenglandandwales/census2021

[4] Evans N. (2007). Muslim pupils urged to boycott school meals. Available at: https://www.lancashiretelegraph.co.uk/news/1499925.muslim-pupils-urged-boycott-school-meals/

[5] Palmer S. (2013). Halal meat in schools. Available at: https://www.whatdotheyknow.com/request/halal_meat_in_schools_16?unfold=1

[6] Department for Education. (2023). School food standards: resources for schools. Available at: https://www.gov.uk/government/publications/school-food-standards-resources-for-schools

[7] Spence S Matthews JN White M Adamson AJ. A repeat cross-sectional study examining the equitable impact of nutritional standards for school lunches in England in 2008 on the diets of 4-7y olds across the socio-economic spectrum. Int J Behav Nutr Phys Act. (2014) 11:128. doi: 10.1186/s12966-014-0128-6, PMID: 25342153 PMC4228190

[8] Stevens L Nelson M. The contribution of school meals and packed lunch to food consumption and nutrient intakes in UK primary school children from a low income population. J Hum Nutr Diet. (2011) 24:223–32. doi: 10.1111/j.1365-277X.2010.01148.x, PMID: 21332839

[9] Evans CEL Cleghorn CL Greenwood DC Cade JE. A comparison of British school meals and packed lunches from 1990 to 2007: meta-analysis by lunch type. Br J Nutr. (2010) 104:474–87. doi: 10.1017/S0007114510001601, PMID: 20500928

[10] Parnham JC Laverty AA Majeed A Vamos EP. Half of children entitled to free school meals did not have access to the scheme during COVID-19 lockdown in the UK. Public Health. (2020) 187:161–4. doi: 10.1016/j.puhe.2020.08.019 32980783 PMC7447260

[11] Newcastle City Council. (2021). School meals. Available at: https://www.newcastle.gov.uk/services/schools-learning-and-childcare/parent-information/school-meals

[12] Coulson H Milbourne P. Food justice for all?: searching for the ‘justice multiple’ in UK food movements. Agric Hum Values. (2021) 38:43–58. doi: 10.1007/s10460-020-10142-5

[13] UK Parliament. (2014). Social justice strategy. Available at: https://hansard.parliament.uk/Lords/2014-10-16/debates/14101679000329/SocialJusticeStrategy

[14] Asri R. (2022). What is halal food? Meaning, types and halal meat certification. Available at: https://www.honestfoodtalks.com/what-is-halal-food/

[15] Yakar EE. The progress of halal food trend in the United Kingdom. J Interdiscip Food Stud. (2021) 1:1–18. doi: 10.5281/zenodo.5791121

[16] Dalton J. (2018). Council becomes first in UK to ban unstunned halal meat for schools, in move branded 'Islamaphobic'. Available at: https://www.independent.co.uk/news/uk/home-news/no-stun-halal-meat-ban-lancashire-schools-pupils-muslim-islamophobic-a8445896.html

[17] Hong J. (2021). Not on the menu: halal, kosher options limited in California school lunches. Available at: https://calmatters.org/education/k-12-education/2021/10/california-school-lunch-halal-kosher/

[18] 5Pillars. (2023). Wakefield primary school served pupils haraam meat labelled 'halal'. Available at: https://5pillarsuk.com/2023/07/10/wakefield-primary-school-served-pupils-haraam-meat-labelled-halal/

[19] Jasbir A (2013). Birmingham school says sorry for serving non-halal meat to Muslim pupils. Available at: https://www.birminghammail.co.uk/news/local-news/birmingham-school-sorry-serving-non-halal-1330696

[20] Terrell J. (2023). None of the Muslim kids can eat': Illinois to provide halal and kosher meals to schoolkids. Available at: https://www.theguardian.com/environment/2023/jun/01/illinois-halal-kosher-school-meals

[21] Story M Neumark-Szainer D French S. Individual and environmental influences on adolescent eating behaviors. J Am Diet Assoc. (2002) 102:S40–51. doi: 10.1016/S0002-8223(02)90421-9 11902388

[22] Ryan G. Introduction to positivism, interpretivism and critical theory. Nurse Res. (2018) 25:14–20. doi: 10.7748/nr.2018.e1466 29546962

[23] Sada AN Maldonado A. Research methods in education. Sixth edition - by Louis Cohen, Lawrence Manion and Keith Morrison. Br J Educ Stud. (2007) 55:469–70. doi: 10.1111/j.1467-8527.2007.00388_4.x

[24] Braun V Clarke V. Using thematic analysis in psychology. Qual Res Psychol. (2006) 3:77–101. doi: 10.1191/1478088706qp063oa

[25] Boyatzis RE. Transforming qualitative information: thematic analysis and code development. Thousand Oaks, California: Sage (1998).

[26] Glaser B Strauss A. Discovery of grounded theory: strategies for qualitative research. New York: Routledge (2017).

[27] Lancashire City Council. (2018). Lancashire County Council's policy on the supply of halal meat to schools consultation 2018 summary report. Available at: https://council.lancashire.gov.uk/mgConvert2PDF.aspx?ID=136648

[28] United Nations Educational Scientific and Cultural Organisation. (2005). Convention on the protection and promotion of the diversity of cultural expressions. Available at: https://en.unesco.org/about-us/legal-affairs/convention-protection-and-promotion-diversity-cultural-expressions

[29] Evans CEL Harper CE. A history and review of school meal standards in the UK. J Hum Nutr Diet. (2009) 22:89–99. doi: 10.1111/j.1365-277X.2008.00941.x, PMID: 19302115

[30] Pickett W Michaelson V Davison C. Beyond nutrition: hunger and its impact on the health of young Canadians. Int J Public Health. (2015) 60:527–38. doi: 10.1007/s00038-015-0673-z 25929577 PMC4480846

[31] Lalli GS. Schools, food and social learning. New York: Routledge (2019).

[32] Clarke J Fletcher B Lancashire E Pallan M Adab P. The views of stakeholders on the role of the primary school in preventing childhood obesity: a qualitative systematic review. Obes Rev. (2013) 14:975–88. doi: 10.1111/obr.12058, PMID: 23848939

[33] Wu XY Zhuang LH Li W Guo HW Zhang JH Zhao YK. The influence of diet quality and dietary behavior on health-related quality of life in the general population of children and adolescents: a systematic review and meta-analysis. Qual Life Res. (2019) 28:1989–2015. doi: 10.1007/s11136-019-02162-4, PMID: 30875010

[34] Day RE Sahota P Christian MS Cocks K. A qualitative study exploring pupil and school staff perceptions of school meal provision in England. Br J Nutr. (2015) 114:1504–14. doi: 10.1017/S0007114515002834, PMID: 26329922

[35] Devine LD Hill AJ Gallagher AM. Improving adolescents' dietary behaviours in the school-setting: challenges and opportunities. Proc Nutr Soc. (2023) 82:172–85. doi: 10.1017/S0029665123002197 36916515

[36] Gilmour A. Gill S. Loudon G. (2021). Perspectives of UK catering staff on adolescents’ food choices at school. Available at: https://schoolnutrition.org/uploadedFiles/5_News_and_Publications/4_The_Journal_of_Child_Nutrition_and_Management/Spring_2021/Perspectives-of-UK-Catering-Staff-on-Adolescents-Food-Choices-at-School-Spring2021.pdf.

[37] Kracht CL Burkart S Flanagan EW Melnick E Luecking C Neshteruk C. Policy, system, and environmental interventions addressing obesity and diet-related outcomes in early childhood education settings: a systematic review. Obes Rev. (2023) 24:e13547. doi: 10.1111/obr.13547, PMID: 36601716 PMC10214414

[38] Twiner A Cook G Gillen J. Overlooked issues of religious identity in the school dinners debate. Camb J Educ. (2009) 39:473–88. doi: 10.1080/03057640903352457

[39] Oostindjer M Aschemann-Witzel J Wang Q Skuland SE Egelandsdal B Amdam GV. Are school meals a viable and sustainable tool to improve the healthiness and sustainability of children’s diet and food consumption? A cross-national comparative perspective. Crit Rev Food Sci Nutr. (2017) 57:3942–58. doi: 10.1080/10408398.2016.1197180 27712088

